# Boronic Acid as Glucose-Sensitive Agent Regulates Drug Delivery for Diabetes Treatment

**DOI:** 10.3390/ma10020170

**Published:** 2017-02-13

**Authors:** Li Zhao, Qiongwei Huang, Yangyang Liu, Qing Wang, Liyan Wang, Shanshan Xiao, Fei Bi, Jianxun Ding

**Affiliations:** 1 Laboratory of Building Energy-Saving Technology Engineering, College of Material Science and Engineering, Jilin Jianzhu University, Changchun 130118, China; zhaolizdl@163.com (L.Z.); wlynzy@163.com (L.W.); xiaoshanshan@jlju.edu.cn (S.X.); bifei1224@163.com (F.B.); 2 Department of Endocrinology, China-Japan Union Hospital of Jilin University, Changchun 130033, China; huangqw0870@mails.jlu.edu.cn (Q.H.); wangqing5151@126.com (Q.W.); 3 Department of Endocrine, the First Affiliated Hospital to Changchun University of Chinese Medicine, Changchun 130117, China; yyliu_cucm@hotmail.com; 4 Key Laboratory of Polymer Ecomaterials, Changchun Institute of Applied Chemistry, Chinese Academy of Sciences, Changchun 130022, China

**Keywords:** phenylboronic acid, glucose-sensitivity, drug delivery, diabetes therapy

## Abstract

In recent years, glucose-sensitive drug delivery systems have attracted considerable attention in the treatment of diabetes. These systems can regulate payload release by the changes of blood glucose levels continuously and automatically with potential application in self-regulated drug delivery. Boronic acid (BA), especially phenylboronic acid (PBA), as glucose-sensitive agent has been the focus of research in the design of glucose-sensitive platforms. This article reviews the previous attempts at the developments of PBA-based glucose-sensitive drug delivery systems regarding the PBA-functionalized materials and glucose-triggered drug delivery. The obstacles and potential developments of glucose-sensitive drug delivery systems based on PBA for diabetes treatment in the future are also described. The PBA-functionalized platforms that regulate drug delivery induced by glucose are expected to contribute significantly to the design and development of advanced intelligent self-regulated drug delivery systems for treatment of diabetes.

## 1. Introduction

Diabetes, a human disorder of glucose metabolism, has been one of the globally leading causes of death with a sharply increasing number of patients. The total number of diabetes patients will be about 366 million all over the world in 2030, among which 42 million will be in China as predicted by the World Health Organization (WHO) [1]. Currently, the treatment for diabetes relies on the multiple daily exogenous insulin injections to persistently regulate blood glucose levels. However, the frequent insulin injections cause long-term complications with poor patient compliance. With regards to this, great efforts have been devoted to investigating self-regulated insulin delivery systems, which would be a promising alternative to control the diabetic blood glucose levels. The self-regulated insulin delivery systems could deliver an accurate dose of insulin upon the changes of blood glucose concentration, mimicking the biofeedback system. Three kinds of glucose-sensitive materials, based on boronic acid (BA, particularly phenylboronic acid (PBA)), glucose oxidase (GOD), and concanavalin A (Con A), have attracted growing attention, which are utilized in self-regulated insulin delivery systems [2,3,4,5,6]. Among them, PBA and its derivatives have shown more potential applications in glucose-sensitive insulin self-regulated delivery systems due to the selective glucose-sensitivity, versatile design, greater stability, and long term storability than protein-based systems (i.e., GOD and Con A) [7,8,9].

**Figure 1:**
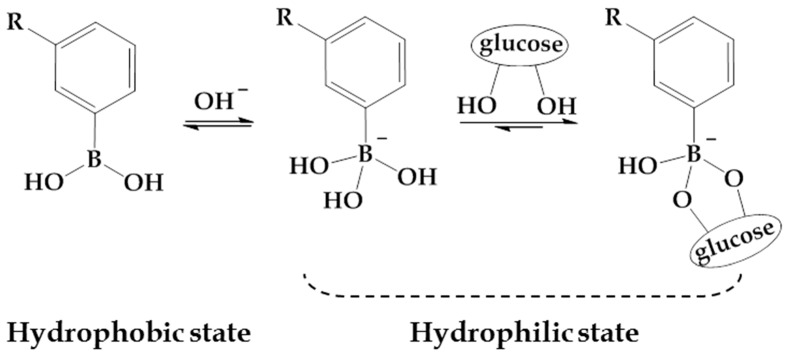
Illustrational scheme of reversible binding of glucose to PBA.

It is well-known that PBA and its derivatives exhibit higher affinities with *cis*-diol compounds through reversible boronate formation, endowing the PBA-functionalized materials with promising applications in the glucose-sensitive drug delivery. In aqueous solution, there are two forms of PBA moieties, and there is an equilibrium between them (Figure 1). The neutral trigonal-planar form is relatively hydrophobic, while the negatively charged tetrahedral boronate form is relatively hydrophilic. In a weak basic solution at pH above the p*K*_a_ of PBA (i.e., 8.2–8.6), the trigonal-planar and hydrophilic PBA can be charged by combining with –OH to form relative hydrophilic moiety, which is well known to selectively combine with *cis*-diol compounds through the formation of stable cyclic boronic ester [10,11]. Even though the neutral trigonal-planar form also could form complexes with diols, the formation of cyclic boronic ester between charged and hydrophilic PBA and diols is more stable. It makes the ionization equilibrium shift to the hydrophilic and negatively charged PBA, further improving the hydrophilicity of PBA-containing materials [12,13]. The increase of the hydrophilicity lays the foundation for the glucose-sensitivity of PBA-functionalized materials. Glucose, one kind of *cis*-diol compound, is well-documented to form stable glucose-PBA complex at neutral or alkaline pH. When glucose is added, the increase of hydrophilicity of PBA-containing carriers provides the swelling or disassembling of the drug delivery vehicles, resulting in glucose-triggered drug release. Introduction of PBA into polymers can endow the PBA-functionalized materials with great glucose-sensitivity, which has potential candidates for self-regulated drug delivery [14,15].

## 2. PBA-Functionalized Materials and Polymers

The glucose-responsive formulation with PBA as a sensitive element, greatly utilized in the design of self-regulated insulin delivery systems for the treatment of diabetes, has attracted growing scientific attention. Many materials and polymers are functionalized by PBA to fabricate glucose-sensitive platforms with macroscopic, microscopic, or nanoscopic sizes. There are great developments in the glucose-sensitive carriers based on PBA for self-regulated drug delivery. Also, this prospective study provides the major challenges and opportunities of PBA-based glucose-sensitive drug delivery.

Two methods were proposed to prepare the PBA-functionalized materials. One is the polymerization of PBA-functionalized monomers, and the other is the modification of materials with PBA [16,17]. Poly(methacrylic acid) (PMAA) and its derivatives functionalized by PBA are usually utilized as the glucose-sensitive materials due to the good biocompatibility and facile modification. The pH- and glucose-sensitive poly(acrylic acid) (PAA)-contained hydrogels based on PBA could be prepared and used as drug delivery vehicles. These hydrogels, physically or chemically cross-linked to maintain their structural integrity, are soft materials with remarkable compatibility with blood and tissue [18,19]. The glucose sensitivity of the hydrogels is due to the changes of hydrophilicity of PBA moiety. In the presence of glucose, the PAA polymers containing PBA alter their swelling properties due to phase changes, hydrophobic–hydrophilic interchanges, or ionizations with the preloaded drug release [20]. The temperature and glucose dual-sensitive PAA-based hydrogels containing PBA and poly(*N*-isopropylacrylamide) (PNIPAM) have been studied for uses in glucose detection, drug delivery, and diagnosis [21]. PNIPAM, a well-known thermo-sensitive material, can reversibly switch between a swollen and a shrunken state with temperature changes. The volume phase transition temperature (VPTT) of PNIPAM is about 32 °C, which could be adjusted to physiological temperature (i.e., 37 °C) via the change of compositions of PNIPAM-based copolymers [22,23]. Using the temperature-sensitivity, drugs could be loaded into the vehicles with the enhanced glucose-sensitivity at physiological pH and temperature [24].

In addition, the PAA materials containing PBA could also be prepared to microcosmic and nanoscopic carriers with good glucose-sensitive profiles. The glucose-sensitive microgels and microcapsules based on PAA and PBA have potential applications in the treatment of diabetes [25,26,27,28,29]. Ravaine and coworkers synthesized the monodisperse submicrometric PNIPAM microgels modified with 3-acrylamidophenylboronic acid (AAPBA) and studied the swelling of microgel with different PBA contents at different temperatures with or without glucose [30]. The incorporation of AAPBA resulted in a decrease of VPTT compared to that of the native PNIPAM particles. It expected that the swelling response increased with increasing receptor density. However, at a constant glucose concentration, the reached swollen state was also strongly dependent on the initial temperature of the suspension. When the initial temperature > VPTT, the particles was shrunk and the swelling was slight, which could be explained in that glucose could not diffuse inside the particle when it was initially collapsed. In this case, only a small amount of glucose could link to the PBA incorporated on the particles. Whereas, a slight swelling of the particles was necessary to allow permeation and molecular diffusion in the internal of the particle, when the initial temperature < VPTT. In such conditions, PBA could be linked to form a more hydrophilic boronic ester, leading to a much higher swelling degree.

The glucose-sensitive microgel monolayer films were fabricated on both silicon wafers and quartz slides, and the microgel particles were highly monodisperse with an average diameter of 540 nm [31]. Alizarin Red S (ARS) and fluorescein isothiocyanate (FITC)-insulin were separately loaded in the monolayers, and their release kinetics under various conditions were measured. At low temperature, the drug release followed a passive diffusion mechanism, but with increasing temperature, the drug was released mainly by a squeeze-out mechanism depending on whether there was binding between drug and PBA group through a reversible phenylboronate ester bond. The drug release profiles would guide the design of new self-regulated insulin release systems based on P(NIPAM-PBA) microgels.

Layer-by-layer (LbL) technique is usually used to fabricate thin films and capsules with controlled composition and thickness [32]. A novel polyelectrolyte, containing PBA as a glucose-sensitive moiety has been synthesized and used for the fabrication of glucose-sensitive hollow polyelectrolyte capsules using the LbL technique [33]. The polyelectrolyte capsule was prepared using 3-acrylamidophenylboronic acid-dimethyl aminoethyl acrylate copolymer (PAD) with PBA unit as a polycation and 4-sodium polystyrene sulfonate (PSS) as a polyanion. The permeability of the capsules to the FITC-labeled bovine serum albumin (FITC-BSA) was investigated and the result showed that as the capsule only decomposed at glucose concentrations above 2.5 mg/mL, they remained intact at normal (i.e., healthy) glucose levels (buffered at pH 9).

Glucose-sensitive nanoscale platforms based on PAA and PBA, such as micelle and nanogel, have attracted more interest and have more promising applications due to great glucose-sensitivity, long-term storability, and good biocompatibility [34].

The micelle was prepared by the self-assembly of PBA-functionalized amphiphilic copolymer poly(ethylene glycol)-*block*-poly(acrylic acid-*co*-acrylamidophenylboronic acid) (PEG-*b*-P(AA-*co*-AAPBA)) [35]. The core was hydrophobic and composed of PAAPBA, whereas the shell was hydrophilic and composed of PEG. In aqueous solution at pH = 7.4, the core-shell structure disaggregated as it was exposed to glucose. Hollow nanogel composed of PNIPAm and poly(*N*-phenylboronic acid acrylamide) (PPBAA) was prepared by two-step colloidal template polymerization [36]. PNIPAM and PPBAA endowed the nanogel with temperature-sensitivity and glucose-responsive properties, respectively.

In addition to PAA, the PBA-functionalized hybrid materials are also used as glucose-sensitive drug carriers. Silicon and its oxides, especially mesoporous silica materials (MSMs) are widely used as biomaterials due to the biocompatible and modifying properties [37,38]. Mesoporous silica nanoparticles, with large capacity for drug loading, tailorable mesoporous structure, and modification properties, have attracted intense interest for use as glucose-sensitive self-regulated drug delivery carriers [39,40,41]. The MSN-based double-drug delivery system was designed to control the release of gluconic acid-modified insulin (G-Ins) and cyclic adenosine monophosphate (cAMP) triggered by glucose. cAMP could enhance stimulated insulin secretion via activing the Ca^2+^ channels of pancreas beta cells [42]. cAMP was encapsulated inside MSN with immobilization of G-Ins on the exterior surface of PBA-functionalized MSN. When in glucose solution, the PBA-glucose complexes made the surface of PBA-functionalized MSN hydrophilic resulting into the release of G-Ins, and the release of G-Ins opened the channel of MSN with the release of cAMP. The good biocompatibility and cell uptake properties of nanodevice make the PBA-functionalized MSN a promising glucose-sensitive insulin-release system.

Even though the PBA-functionalized PAA and MSM are well biocompatible and glucose-sensitive, the non-degradability limits their further application because of the difficulty for blood clearance. Polysaccharides, containing amount of acetylglucosamine and glucosamine (sugar) units, are widely used in various biomedical areas, such as tissue engineering and drug delivery [43,44]. Polysaccharides used for glucose-sensitive drug delivery have been the subject of intense research. Chitosan (CS), a kind of natural polysaccharide, is composed of glucosamine and *N*-acetyl-glucosamine, and the primary amino groups are quite active, which could react with various groups [45,46]. The PBA-grafted chitosan (PBACS) was fabricated by the coupling reaction of the amino group in AAPBA and the carboxyl group in *N*-(carboxyacyl) chitosan, and the PBACS nanoparticle could be used to load insulin. In glucose solution, the insulin release presented a glucose concentration-dependent manner because more glucose-PBA complexes formed with enhanced hydrophilicity of the nanoparticle when glucose concentration increased. The released insulin had the original tertiary structure compared with that of standard insulin [47]. The glucose-responsive nanocapsule based on CS and PBA was readily fabricated by LbL technique [48]. Using silicon dioxide (SiO_2_) as a template, chitosan-*N*-acetyl-l-cysteine conjugate (CS-NAC) and glycopolymer poly(d-gluconamidoethyl methacrylate-*r*-3-acrylamidophenylboronic acid) P(GAMA-*r*-AAPBA) was alternatively added to the particles resulting in multilayered polyelectrolyte hybrid shell after etching the amino-functionalized SiO_2_ by ammonium fluoride (NH_4_F)/hydrogen fluoride (HF). The polyelectrolyte nanocapsules could control insulin release induced by glucose with a potential application in self-regulated drug delivery. The glucose-sensitive core-shell nanocarriers also could be prepared by using mesoporous silica nanoparticles coated with dextran-maleic acid (Dex-Ma) and then grafted with AAPBA [49]. In addition, the glucose-sensitive drug carriers based on PBA also could be prepared via the complexations of PBA-functionalized materials and ortho hydroxyl groups in Dex or CS [50,51,52]. The PBA-functionalized glycopolymer nanoparticles for biomacromolecules delivery were also investigated [53,54,55,56].

Poly(amino acid) or its derivatives, one kind of important biocompatible and biodegradable polymers, are widely used as biomedical materials due to their availability of side functional groups [57,58,59,60,61]. Synthetic polypeptides, especially poly(l-glutamic acid), poly(l-aspartic acid) and poly(l-lysine), could be modified due to the reactive side groups and could self-assemble into micelles, vesicles, and solid nanoparticles with controlled drug delivery capabilities. Compared with PAA, the PBA-functionalized polypeptides have promising applications in the self-regulated drug delivery systems because of excellent biodegradability. Chen and coworkers prepared the polypeptide micelles using methoxy poly(ethylene glycol)*-block*-poly(l-glutamic acid-*co*-*N*-3-l-glutamylamidophenylboronic acid) (mPEG-*b*-P(GA-*co*-GPBA)) [62], and the polypeptide micelles have good glucose-triggered insulin release profiles [63]. In addition, the same group designed a novel polypeptide nanogel that was prepared by glycopolypeptides poly(ethylene glycol)-*block*-poly-(γ-benzyl-l-glutamate-*co*-(γ-propargyl-l-glutamate-*graft*-glucose) (mPEG-*b*-P(BLG-*co*-(PLG-*g*-Glu))) using adipoylamidophenylboronic acid as a cross-linker [64]. The excellent glucose-sensitivity of polypeptide nanogel associated with competitive binding mechanism. That is, glucose in solution could complex with PBA substituting the conjugated glucose on polypeptide. In addition, Shi and coworkers reported a glucose-responsive complex polymer micelle, which was prepared by the self-assembly of two types of diblock copolymers, poly(ethylene glycol)-*block*-poly(aspartic acid-*co*-aspartamidophenylboronic acid) (PEG-*b*-P(Asp-*co*-AspPBA)) and poly(*N*-isopropylacrylamide)-*block*-poly(aspartic acid-*co*-aspartamidophenylboronic acid) (PNIPAM-*b*-P(Asp-*co*-AspPBA)) [65]. Also, through the self-assembly of these two polypeptides, polymer vesicles with good glucose-sensitivity could be obtained [66]. In addition, via a template of α-cyclodextrin (α-CD)/PEG inclusion complex, polymer vesicles based on the complexation between glucosamine (GA)-containing block copolymers of poly(ethylene glycol)-*block*-poly(aspartic acid-*co*-aspart-glucosamine) (PEG_45_-*b*-P(Asp-*co*-AspGA)) and PEG_114_-*b*-P(Asp-*co*-AspPBA) could be fabricated [67]. A novel double-layered nanogel of glycol chitosan (GC)/sodium alginate (SA)-poly(l-glutmate*-co*-*N*-3-l-glutamylphenylboronic acid) (PGGA) graft copolymer (GC/SA-PGGA) may be a promising candidate used as a self-regulated drug delivery system for the therapy of diabetes, resulting from the excellent glucose-recognition insulin release profile [68].

Overall, PAA and its derivatives functionalized by PBA are important biocompatible materials used in glucose-sensitive self-regulated drug delivery systems. In addition, the PBA-functionalized hybrid materials and polysaccharides are also widely used as glucose-sensitive drug carriers. For the therapy of diabetes, long-term injection of insulin is needed. Therefore, the biocompatible and biodegradable polymers, such as polypeptides, have attracted more interest for the design of glucose-sensitive self-regulated drug delivery systems. All of these platforms with successfully realized glucose-induced drug release profiles may have promising potential application in the treatment of diabetes.

## 3. Glucose-Sensitivity

**Figure 2:**
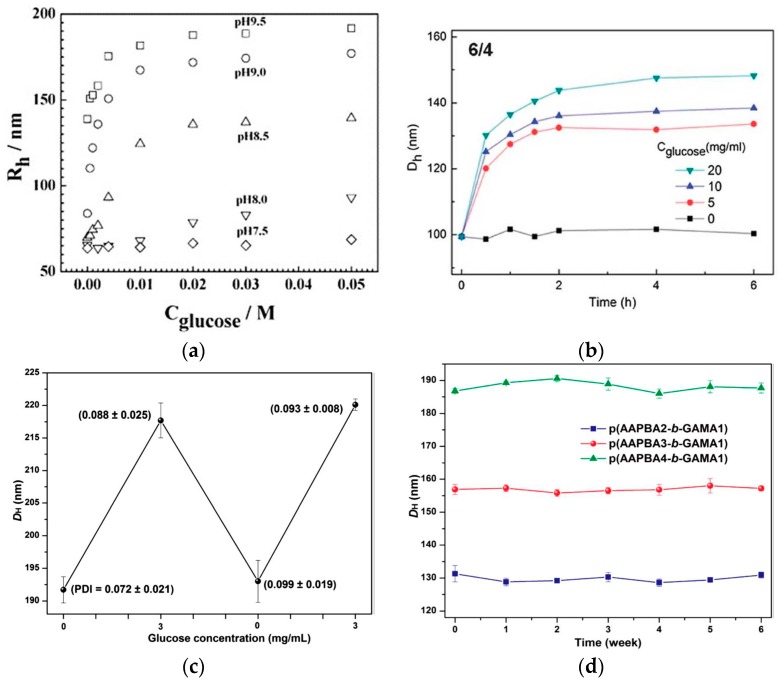
(**a**) *R*_h_ values of P(NIPAM−PBA) microgel (10.0 mol % PBA) as a function of glucose concentration, measured in 0.005 M PBs of different pH values at *T* = 25 °C; (**b**) time-dependent *D*_h_ of 6/4 complex polymer micelle under various glucose concentrations; (**c**) reversible glucose-sensitivity of P(AAPBA4-*b*-GAMA1) nanoparticle; (**d**) the stability of blank p(AAPBA-*b*-GAMA) NP in pH 7.4 PBS. (Reprinted from Refs. 71, 65, and 8.)

Ideal glucose-sensitive self-regulated drug delivery systems could control insulin release at physiological pH and temperature triggered by diabetic blood glucose level. More importantly, the self-regulated drug delivery system can maintain high insulin bioavailability and supply precise glycemic control with good patient compliance. However, the glucose-sensitive profiles have been mostly studied in the alkaline release medium due to the p*K*_a_ of PBA (8.2–8.6). PBA moiety is Lewis acid, and can ionize and form stable cyclic boronic ester only when the pH of solution is above the p*K*_a_ of PBA. Therefore, the glucose-sensitivity for the PBA-functionalized delivery vehicle could be presented in relative basic solution [22,69,70]. As shown in Figure 2a, the PNIPAM-based microgels functionalized with PBA groups was glucose-sensitive under basic conditions in the pH range of 8.0–9.5, while at pH = 7.5 the glucose-sensitivity of the microgels was negligible at different glucose concentrations [71]. Great efforts have been devoted to decrease the p*K*_a_ value of PBA derivatives to enhance complexation between glucose, sugar units on polysaccharides or glycopolymers, and PBA moiety to obtain the glucose-sensitivity at physiological pH [72]. The introduction of electron-withdrawing groups—such as carbonyl, nitro, and halogen—into the phenyl ring could adjust the glucose-sensitive pH ranges [73,74]. Furthermore, the incorporation of an amine adjacent to the PBA also could reduce the p*K*_a_ value of PBA derivatives, endowing the PBA-functionalized vehicles with glucose-sensitivity at physiological pH [75,76,77]. As we all know that the prandial blood glucose concentration for diabetics is above 2.0 mg·mL^−1^, while the prandial blood glucose level in the healthy human body is below 1.5 mg·mL^−1^ (1.0 mg·mL^−1^ = 5.56 mM) [78]. The ideal glucose-sensitive PBA-modified drug platforms should release an amount of drug when the glucose concentration is equal to or above 2.0 mg·mL^−1^, while having no or slight drug release under healthy blood glucose [14]. However, many studies reported that the glucose-sensitive drug carriers had glucose-sensitivity at much higher glucose concentrations than normal blood glucose levels as shown in Figure 2b [65,79]. By adjustment of the PBA contents with intelligent structure design, excellent glucose-sensitive drug delivery carriers have been exploited [34,80,81].

Using LbL technique, the glucose-sensitive polyelectrolyte nanocapsule CS-NAC/P(GAMA-*r*-AAPBA) was fabricated with a synthesized random glycopolymer poly(d-gluconamidoethyl methacrylate-*r*-3-acrylamidophenylboronic acid) (P(GAMA-*r*-AAPBA)), containing PBA as a glucose-sensitive moiety and modified CS as a polycation [48]. The nanocapsules had good glucose-sensitivity at 1.5 mg·mL^−1^ glucose concentration, and the glucose-sensitivity was reversible at physiological pH. Nanoparticles, prepared by the cross-linking between the diol groups of carbohydrates and PBA on glycopolymer poly(d-gluconamidoethyl methacrylate-*block*-3-acrylamidophenylboronic acid) (P(GAMA-*b*-AAPBA)), also had excellent reversible glucose sensitivity (Figure 2c) [8]. In addition, the stability of the carriers in aqueous solution is also very important for the promising application in self-drug delivery systems (Figure 2d) [8,52].

The glucose-sensitivity of PBA-based drug delivery platforms at physiological pH is very important for the design of self-regulated drug delivery systems. More importantly, whether the glucose-sensitivity is reversible and precise at the fluctuating range of blood glucose in diabetic patients restricts the promising application for the clinical therapy of diabetes.

## 4. Glucose-Triggered Drug Delivery

**Figure 3:**
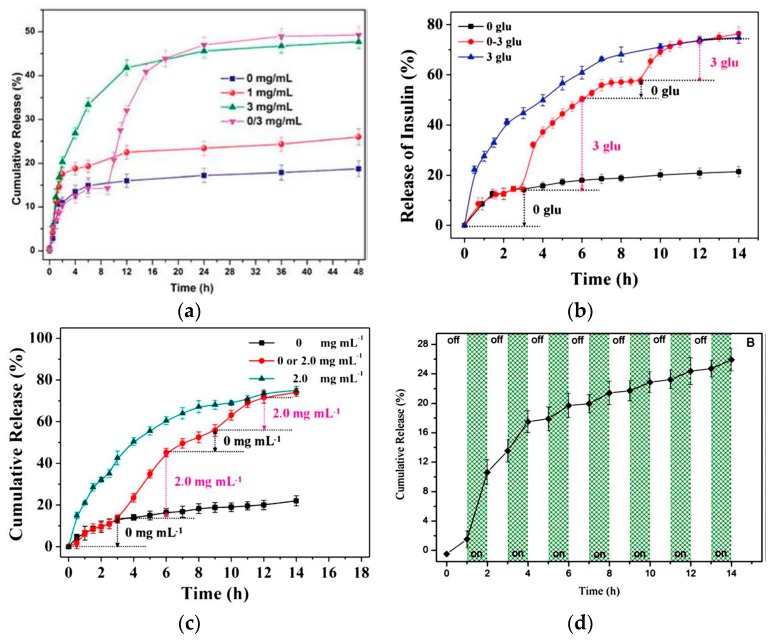
(**a**) In vitro cumulative release of insulin in pH 7.4 PBS from p(AAPBA4-*b*-GAMA1) nanoparticle at various glucose concentrations (0, 1.0, and 3.0 mg·mL^−1^), and medium only for the first 12 h and then 3.0 mg·mL^−1^; (**b**) glucose-sensitive insulin release from micelle in PBS with alternating glucose concentrations (0 or 3.0 mg·mL^−1^ marking as 0 glu and 3 glu, respectively, while the marker of 0–3 glu represented the cumulative insulin release in PB with alternant 0 or 3.0 mg·mL^−1^ glucose) in PBS at pH 7.4, 37 °C; (**c**) glucose-sensitive ARS release from polypeptide nanogel in PBS with alternating glucose concentrations (0 or 2.0 mg·mL^−1^) at 37 °C; (**d**) glucose-triggered on–off release of insulin from polymer vesicles. (Reprinted from Refs. 8, 63, 82, and 66.)

The glucose-sensitive drug carriers based on PBA release payload induced by glucose, and the ideal glucose-sensitive drug carriers should possess on–off release profiles. In details, amount of payload is released quickly when the glucose concentration is above diabetic blood glucose levels (above 2.0 mg·mL^−1^ glucose), with no or slight drug release under healthy blood glucose level (1.0 mg·mL^−1^ glucose). When the concentration of glucose in the medium increased, more payload was released because more glucose could complex with PBA to form boronate esters with the enhanced hydrophilicity of the platforms (Figure 3a) [8]. The alternant drug release ability is one of most important requisites for potential clinical-use glucose-sensitive drug delivery systems, which could achieve on-demand drug release. The mPEG-*b*-P(GA*-co*-GPBA) micelles possessed alternant glucose-sensitive insulin release in phosphate buffer (PB) [63]. As shown in Figure 3b, the release of insulin was slow in the medium without glucose and the cumulative amount of insulin had a slight increase after 3 h. Between the micellar core and insulin molecule there were hydrophobic, electrostatic, and hydrogen bonding interactions, which tightly wrapped insulin in the hydrophobic micellar core. The release of insulin was quick and obvious in the 3.0 mg·mL^−1^ glucose because that more glucoses complexed with PBA, and the formation of boronate esters enhanced the hydrophilicity of the micelles. The enhanced hydrophilicity made micelles swell, and as a result more insulin was released in 3.0 mg·mL^−1^ glucose solution. In the alternant glucose-sensitive insulin release test, the insulin release from micelles was conducted in PB containing 0 or 3.0 mg·mL^−1^ glucose at an interval of 3 h. Only 12.6% of insulin was released at the first 3 h, while when the sample was transferred into the 3.0 mg·mL^−1^ glucose medium in the second 3 h, about 37.7% of insulin was released. When the sample was switched back to PB without glucose, the release of insulin was interrupted because no more glucose-PBA complexes could induce the swelling of micelles. However, the release behavior could be recovered when the insulin-loaded polypeptide micelles were switched back to 3.0 mg·mL^−1^ glucose medium. The glucose-sensitive polypeptide micelles that undergo glucose-switchable release show promising application in self-regulated drug delivery. The nanogel prepared by one-pot thiol-ene copolymerization of pentaerythritol tetra(3-mercaptopropionate) (QT), poly(ethylene glycol) diacrylate (PEGDA), poly(ethylene glycol) acrylate (mPEGA), and AAPBA also had on-off drug release profiles (Figure 3c) [82]. The on-off regulation of drug release offers an effective strategy for self-regulated insulin delivery in response to glucose, and the repeated on-demand drug delivery restricts the clinical application of glucose-sensitive carriers for therapy of diabetes (Figure 3d) [66].

**Figure 4:**
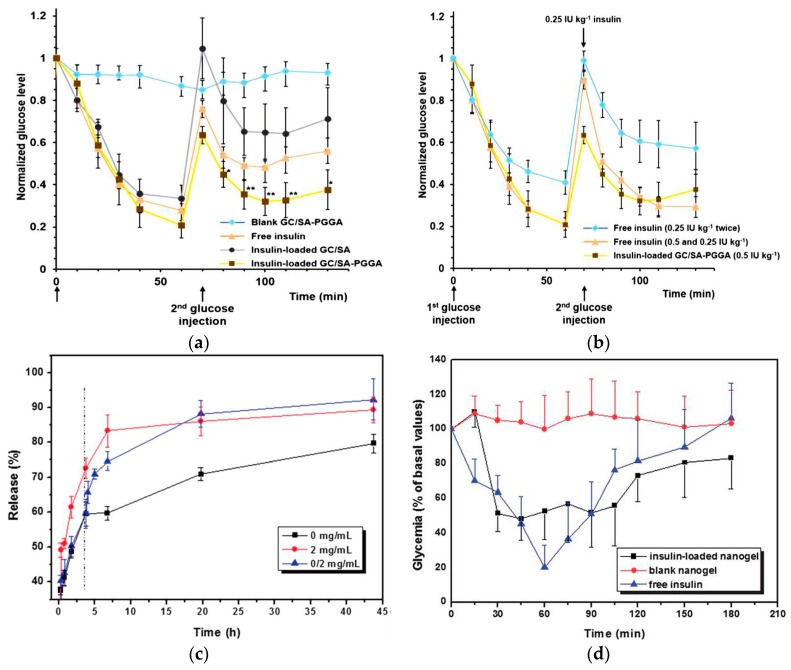
(**a**) Normalized blood glucose levels in mice by retro-orbital administration of blank GC/SA-PGGA nanogel, free insulin (0.5 IU·kg^−1^), insulin-loaded GC/SA (0.5 IU·kg^−1^), and insulin-loaded GC/SA-PGGA nanogel (0.5 IU·kg^−1^); (**b**) normalized blood glucose levels in mice by administration of free insulin twice (0.25 IU·kg^−1^), free insulin (0.5 and 0.25 IU·kg^−1^), and insulin-loaded GC/SA-PGGA nanogel (0.5 IU·kg^−1^); (**c**) in vitro release of insulin from P(NIPAM-Dex-PBA) nanogel in 0.1 M PBS at pH 7.4 with various glucose concentrations (medium only, 2.0 mg·mL^−1^, and medium only for the first 4 h and then 2.0 mg·mL^−1^); (**d**) profiles of glycemia after a subcutaneous administration of free insulin (2.0 IU·kg^−1^), insulin-loaded nanogel (4 IU·kg^−1^) and blank nanogel in fed diabetic rats. (Reprinted from Refs. 68 and 83.)

The intelligent glucose-triggered drug release abilities of different glucose-sensitive platforms promote the application of diabetes treatment. The double-layered nanogel of GC/SA-PGGA was prepared by an isotropic gelation method and electrostatic interaction between GC and SA-PGGA [68]. The glucose-induced insulin release profiles at diabetic glucose levels in vitro were demonstrated. Moreover, the controlled insulin release capability of GC/SA-PGGA nanogel in vivo was also confirmed in mouse studies. C57BL/6 mice aged eight weeks were chosen to investigate the in vivo demonstration of controlled insulin release. The overnight-fasted mice were anesthetized by intramuscular injection of a mixture of Zoletil^®^ (10.0 mg·kg^−1^) and xylazine (11.0 mg·kg^−1^) during the experiment. Glucose (0.3 g·kg^−1^) dissolved in 100.0 μL of phosphate-buffered saline (PBS) was administered by retro-orbital injection to mice to boost their blood glucose levels to diabetic glucose ranges. The mice were divided into six groups with four rats in each group. Four groups were given administrations of the insulin-loaded GC/SA-PGGA nanogel (0.5 IU·kg^−1^), the insulin-loaded GC/SA (0.5 IU·kg^−1^) using blank GC/SA-PGGA and free insulin (0.5 IU·kg^−1^) as negative and positive controls by retro-orbital injection, respectively. Blood samples were collected from the tail veins of mice at various time points to measure the blood glucose level by a glucose meter (ACCU-CHEK Active, Roche Diagnostics GmbH, Mannheim, Germany), and the last time point was 320 min. All of the groups were administered glucose at 0 min, while excluding the blank GC/SA-PGGA group, other groups were administered glucose again at 70 min. As shown in Figure 4a, excluding the blank GC/SA-PGGA group, all the blood glucose levels of the other groups possessed a similar behavior with lowered glucose levels. However, after the second glucose injection, the blood glucose levels of the insulin-loaded GC/SA-PGGA nanogel group were dramatically decreased for a longer time, indicating that the nanogel could control the insulin release with high pharmacological activity to decrease the blood glucose levels. Additionally, one group of mice were administered free insulin (0.25 IU·kg^−1^) by retro-orbital injection twice at 0 and 70 min, and the other group were administered with free insulin at 0 min (0.5 IU·kg^−1^) and 70 min (0.25 IU·kg^−1^) with different doses. As shown in Figure 4b, the insulin-loaded nanogel exhibited a similar blood glucose reduction effect as that of free insulin treatment, which was administered twice. The results illustrated that the double-layered nanogel could stabilize the encapsulated insulin and decrease glucose concentration with reduced injection numbers. Compared to frequent free insulin injection, the administration of double-layered nanogel could enhance the patient compliance. The glucose-recognition insulin release profiles endowed the nanogel with promising application in self-regulated insulin delivery systems for the treatment of diabetes.

An injectable nanogel P(NIPAM-Dex-PBA) with PNIPAM and PAAPBA using Dex-Ma as a cross-linker, was fabricated [83]. The nanogel presented better dispersion and reversible glucose sensitivity under physiological conditions. The glucose-sensitive insulin release profile was related to the Dex content and glucose concentration in the medium. When the glucose was added into the release medium, the nanogel could control the insulin release quickly triggered by glucose (Figure 4c). Most importantly, the considerable hypoglycemic effect of the insulin-loaded nanogel was confirmed. In an in vivo study, male Wistar rats (250–280 g), provided by the laboratory animal center of the Academy of Military Medical Sciences (certificate number, SYCK 2007-004), were administered streptozotocin (STZ, 45 mg·kg^−1^) intravenously to cause a diabetes type I condition. After the induction of diabetes mellitus, animals were divided into three groups with eight rats in each group. Two groups were administered with insulin-loaded MG3 nanogel and regular insulin with the doses of 4.0 IU·kg^−1^ and 2.5 IU·kg^−1^ body weight, respectively. Animals were kept conscious to avoid any influence of anesthesia on the insulin absorption during the experiment. As a control, the third group was administered with blank polymer directly through subcutaneous injection. The fasting blood glucose levels were collected from the tail vein and determined on a Accu-Chek^®^ Sensor instrument (Roche Diagnostics, Indianapolis, IN, USA). As shown in Figure 4d, diabetic rats treated with the insulin-loaded nanogel maintained a low blood glucose level for almost 2 h while the blood glucose of rats treated with blank nanogel maintained close to the baseline over the course of the study period. The blood glucose level of diabetic rats treated with the insulin-loaded nanogel was reduced to 51% of the baseline level demonstrated by in vivo experiment. The insulin-loaded nanogel possessed prolonged glucose-lowering effect through controlling insulin release triggered by blood glucose levels. The biological activity of released insulin was protected from being destroyed by the hostile environment of the body fluid media endowing the insulin-loaded nanogel significant effect on reducing blood glucose. Moreover, the insulin-loaded nanogel could keep blood glucose levels stable without remarkable fluctuations of blood glucose. The insulin-loaded nanogel with prolonged and stable blood glucose reduction effect provides a possibility to use as glucose-sensitive platform for the therapy of diabetes.

The PBA-functionalized drug delivery systems must have excellent glucose-triggered drug release profiles, which possess on-demand drug release with rapidly alternant drug release ability. The alternant drug release ability can regulate the payload release with the response of fluctuating glucose levels, and the on-demand drug release can regulate the accurate dose of drugs to control the blood glucose levels. All of these are very important for the clinical application of glucose-sensitive drug delivery systems. The significant hypoglycemic effects of the glucose-sensitive drug platforms based on PBA demonstrated by animal experiments promote promising application in diabetes treatment.

## 5. Conclusions

We have reviewed the recent developments in glucose-sensitive drug delivery systems based on PBA. The studies of PBA-based glucose-sensitive self-regulated drug delivery platforms were focused on the therapy of diabetes. Many PBA-functionalized polymers and materials were fabricated to achieve the glucose-sensitive controlled drug release. In order to obtain the glucose-sensitivity of drug carriers under physiological conditions of diabetics, a series of efforts has been made. Glucose-sensitivity and glucose-triggered in vitro drug release under physiological pH and hyperglycemic levels has been obtained. Successful deliveries of insulin in vivo processing significant hypoglycemic effects have further promoted the development of glucose sensitive drug delivery systems. However, some of the major problems of these systems must be overcome to achieve their clinical application for the diabetes treatment.

(i) Simple preparation of PBA-based glucose-sensitive drug platforms. These drug platforms must be prepared efficiently and economically in a simple way with repeated and controlled structures for different preparations. The facile and repeatable preparation of glucose-sensitive drug carriers could maintain the properties of the structure and glucose-sensitivity of drug delivery platforms. More importantly, the glucose-triggered controlled drug release and hypoglycemic effects could also be stabilized. These are very useful to promote the application of a glucose-sensitive drug delivery system in clinical diabetic therapy.
(ii) Excellent glucose-sensitivity under physiological pH in diabetic blood glucose levels. Even though there are many methods to decrease the p*K*_a_ value of PBA derivatives to promote the glucose-sensitivity of the carriers under physiological pH, the glucose-induced drug delivery in diabetic blood glucose levels remains a challenge, which restricts clinical application for diabetes treatment.
(iii) Proof of reproducibility of glucose-sensitivity over multiple recycles. The glucose-triggered drug releases depend on the glucose levels and the loading capacity of drug. At the same drug-loading capacity, a high blood glucose level could induce a greater release of insulin, and the released insulin could lower the concentration of glucose in blood. However, the relationship between the further glucose-triggered insulin release and the hypoglycemic effects of released insulin has been studied rarely, which is very important for the long-term treatment of diabetes. How to accurately control the same amount of drug under decreasing drug concentration gradients restricts the application of glucose-sensitive drug delivery.
(iv) Guarantee of the bioactivity of released drug (e.g., insulin). The payload used to reduce the blood glucose level may be degenerated and inactivated during the preparation of the drug-loaded carriers and the drug release process, which has no hypoglycemic effects. The payload must maintain the original bioactivity to guarantee hypoglycemic effects.
(v) Biocompatibility and biodegradability of the carriers. Medical diabetes treatment is a long process needing safe matrices for in vivo application. The matrices must be non-toxic and friendly to the body. Great biocompatible and biodegradable materials, such as polypeptides, should be chosen in the glucose-sensitive self-regulated drug delivery systems. Polypeptides used in the human body could be degraded into small molecules of amino acids which are nontoxic toward humans.

While there are many challenges restricting glucose-sensitive drug delivery systems based on PBA, it is believed that the self-regulated drug delivery system will be promoted and have promising application in diabetes treatment under more continuous in-depth research.
